# Mismatch repair deficiency is a rare but putative therapeutically relevant finding in non-liver fluke associated cholangiocarcinoma

**DOI:** 10.1038/s41416-018-0199-2

**Published:** 2018-10-31

**Authors:** Benjamin Goeppert, Stephanie Roessler, Marcus Renner, Stephan Singer, Arianeb Mehrabi, Monika Nadja Vogel, Anita Pathil, Elena Czink, Bruno Köhler, Christoph Springfeld, Jan Pfeiffenberger, Christian Rupp, Karl Heinz Weiss, Peter Schirmacher, Magnus von Knebel Doeberitz, Matthias Kloor

**Affiliations:** 1 Institute of Pathology, University Hospital Heidelberg, Im Neuenheimer Feld 224, Heidelberg, Germany; 2 Liver Cancer Center Heidelberg (LCCH), Heidelberg, Germany; 3 Diagnostic and Interventional Radiology, Thoraxklinik at University Hospital of Heidelberg, Heidelberg, Germany; 4 Department of Internal Medicine IV, Gastroenterology and Hepatology, University Hospital Heidelberg, Im Neuenheimer Feld 410, Heidelberg, Germany; 5 National Center for Tumor Diseases, Department of Medical Oncology, University Hospital Heidelberg, Heidelberg, Germany; 6 Department of Applied Tumor Biology, Institute of Pathology, University of Heidelberg, Heidelberg, Germany; 7 Department of General Visceral and Transplantation Surgery, University Hospital Heidelberg, Im Neuenheimer Feld 110, Heidelberg, Germany

**Keywords:** Bile duct cancer, Immunotherapy

## Abstract

**Background:**

A major molecular pathway of genetic instability in cancer is DNA mismatch repair deficiency. High-level microsatellite instability (MSI-H) is currently the best predictor of responsiveness towards immune checkpoint blockade. Data about the prevalence of high-level microsatellite instability in cholangiocarcinoma (CCA) has been conflicting.

**Methods:**

We employed a cohort comprising 308 Western-world, non-liver fluke-associated CCAs (159 intrahepatic, 106 perihilar, and 43 distal). We analysed the mononucleotide microsatellite instability marker panel consisting of BAT25, BAT26, and CAT25 and detected MSI-H in 4/308 CCAs (1.3%).

**Results:**

Patients affected by MSI-H CCA had mostly an atypical histomorphology (*p* = 0.004), showed a longer overall survival, although having a high tumour stage, and were of younger age. Correlation analysis of microsatellite instability status with tumour-infiltrating immune cells, MHC I, and PD-L1 expression in the same cholangiocarcinoma cohort showed higher numbers of CD8 + T cells, FOXP3 + regulatory T cells, CD20 + B cells and high or at least moderate MHC I expression levels in MSI-H CCAs.

**Conclusions:**

Even though the overall number of MSI-H CCAs is low, the dismal prognosis of the disease and the therapeutic option of immune checkpoint blockade in the respective patients justify MSI testing of cholangiocarcinoma, particularly in younger patients showing an atypical histomorphology.

## Introduction

The incidence of DNA mismatch repair-deficient, microsatellite-unstable tumours is of particular clinical relevance since it has been shown that DNA mismatch repair-deficient tumours are significantly more responsive to immune checkpoint blockade, particularly using antibodies directed against the immune checkpoint Programmed cell Death protein 1 (PD-1) or Programmed Death-Ligand 1 (PD-L1),^1^ than DNA mismatch repair-proficient tumours. Cholangiocarcinomas are a diverse group of tumours that either arises from the intrahepatic or extrahepatic biliary tree. Three major clinical phenotypes exist: Cholangiocarcinomas of intrahepatic origin and extrahepatic cholangiocarcinomas with perihilar or distal location. Ampullary tumours were not included in this study, as they are of heterogeneous, often intestinal histologic differentiation and clinically and biologically represent a different tumour type. Clinically, cholangiocarcinoma and even biliary tract cancers in general are often treated as one disease, although genetic heterogeneity as well as differences in clinical behavior is nowadays proven.^2^ Most patients with cholangiocarcinoma present with unresectable or metastatic disease. Despite systemic chemotherapy, patients’ prognosis remains poor and to date there are no established molecular targeted therapies tailored to biliary tract cancer.^3^ Molecular events occurring during the development of cholangiocarcinoma are heterogeneous and likely follow a multistep process encompassing alterations of several tumour suppressor genes such as *KRAS* and *TP53*. The genomic spectra of cholangiocarcinoma have been previously nicely depicted.^4^

However, there is still a number of open questions concerning molecular alterations, which are of relevance for the adequate treatment of cholangiocarcinoma patients. Knowledge of the tumour’s DNA mismatch repair deficiency status, with the advent of immune checkpoint blockade therapeutics, is of enormous interest in a plethora of cancer types,^5^ particularly those with poor prognosis following standard therapy regimens such as cholangiocarcinoma. Regarding the frequency of high-level microsatellite-unstable cholangiocarcinoma patients, existing data are conflicting: Previous studies have reported DNA mismatch repair deficiency and high-level microsatellite instability in up to 30% of cases, particularly in liver-fluke-associated cholangiocarcinoma specimens from endemic regions in Thailand.^6^ However, the true percentage of microsatellite-unstable non-liver-fluke-associated cholangiocarcinoma is unclear. Therefore, we aimed to determine frequency and characteristics of high-level microsatellite instability in a large and well-characterised German cohort of cholangiocarcinoma, including all subtypes.

## Materials and methods

### Clinicopathological characteristics of biliary tract cancer patients

Tissue samples from 308 patients (median age 63 years) who underwent bile duct and/or liver surgery at the University Hospital Heidelberg between 1995 and 2010 were included in this study. The cholangiocarcinoma cohort consisted only of adenocarcinomas, including all histologic variants. Resected specimens consisted of 159 intrahepatic cholangiocarcinomas and 149 extrahepatic cholangiocarcinomas (106 perihilar and 43 distal). None of the patients received radio- and/or chemotherapy prior to surgery. Tumours were restaged according to the 8th TNM Classification of Malignant Tumours and classified after the World Health Organization (WHO) tumour classification system (WHO Classification of Tumours of the Digestive System, 4th ed., 2010) by two experienced pathologists (BG and SS). A summary of clinicopathological data is given in Table 1. The use of the tissues for this study was approved by the institutional ethics committee (206/05).

**Table 1:** Clinicopathological data of the cholangiocarcinoma cohort with complete clinicopathological data and correlation to the high-level microsatellite instability status

CCA cohort	Total Number (%)	MSI− Number (%)	MSI+ Number (%)	*p*-value
Number (%)	308 (100.0)	304 (98.7)	4 (1.3)	
Age				
>Median^d^	154 (50.0)	153 (49.7)	1 (0.3)	
<Median	154 (50.0)	151 (49.0)	3 (1.0)	0.37^a^
Sex				
Male	169 (54.9)	167 (54.2)	2 (0.6)	
Female	139 (45.1)	137 (44.5)	2 (0.6)	1.00^b^
CCA subgroups				
iCCA	159 (51.6)	157 (51.0)	2 (0.6)	
pCCA	106 (34.4)	104 (33.8)	2 (0.6)	
dCCA	43 (14.0)	43 (14.0)	0 (0.0)	0.65^c^
Histology^e^ (predominant pattern)				
NOS	257 (83.4)	256 (83.1)	1 (0.3)	
Papillary	21 (6.8)	19 (6.2)	2 (0.6)	
Mucinous	2 (0.6)	2 (0.6)	0 (0.0)	
Intestinal	9 (2.9)	9 (2.9)	0 (0.0)	
Other	19 (6.2)	18 (5.8)	1 (0.3)	**0.004** ^c^
UICC^f^				
UICC 1	40 (13.0)	40 (13.0)	0 (0.0)	
UICC 2	70 (22.7)	70 (22.7)	0 (0.0)	
UICC 3	64 (20.8)	62 (20.1)	4 (1.3)	
UICC 4	81 (26.3)	79 (25.6)	0 (0.0)	
NA	53 (17.2)	53 (17.2)	0 (0.0)	0.36^c^
pT				
T1	75 (24.4)	73 (23.7)	2 (0.6)	
T2	118 (38.3)	117 (38.0)	1 (0.3)	
T3	91 (29.5)	90 (29.2)	1 (0.3)	
T4	24 (7.8)	24 (7.8)	0 (0.0)	0.65^c^
pN				
N0	116 (37.7)	116 (37.7)	0 (0.0)	
N1	138 (44.8)	134 (43.5)	4 (1.3)	
Nx	54 (17.5)	54 (17.5)	0 (0.0)	0.06^b^
M				
M0	298 (96.8)	294 (95.5)	4 (1.3)	
M1	10 (3.2)	10 (3.2)	0 (0.0)	0.71^b^
G				
G1	16 (5.2)	16 (5.2)	0 (0.0)	
G2	214 (69.5)	210 (68.2)	2 (0.6)	
G3	78 (25.3)	78 (25.3)	2 (0.6)	0.41^c^
L				
L0	132 (42.9)	132 (42.9)	0 (0.0)	
L1	176 (57.1)	172 (55.8)	4 (1.3)	0.08^b^
V				
V0	222 (72.1)	218 (70.8)	4 (1.3)	
V1	86 (27.9)	86 (27.9)	0 (0.0)	0.21^b^
Pn				
Pn0	244 (79.2)	240 (77.9)	4 (1.3)	
Pn1	64 (20.8)	64 (20.8)	0 (0.0)	0.30^b^

*NOS* not otherwise specified, i.e., typical acinar, tubular, glandular pancreatobiliary histomorphology.

Significant values are marked in bold. ^a^Mann–Whitney U-test.

^b^Fisher’s exact test

^c^Chi-square test.

^d^Median patients’ age was 63 years.

^e^For all tumours that showed a non-not otherwise specified (NOS) histomorphology the predominant pattern was assessed.

^f^Cases with pNx had no lymph nodes resected, therefore, UICC status could not be assessed

### Tissue microarray construction

From all 308 cholangiocarcinoma formalin-fixed paraffin-embedded tissue blocks, 3 µm sections were cut and stained with haematoxylin and eosin. Representative areas were marked by two experienced pathologists (BG and SS). For each case, tumour tissue cores (1.0 mm diameter) from the selected representative tumour areas were punched out of the sample tissue blocks and embedded into a new paraffin array block using a tissue microarrayer (Beecher Instruments, Woodland, CA, USA).

### Immunohistochemical staining of DNA mismatch repair proteins

Immunohistochemical analyses were performed on 3 μm thick sections in all tumours classified as microsatellite-unstable by polymerase chain reaction-based methods. Briefly, the slides were pretreated by boiling for 10 min with a microwave in target retrieval buffer (pH 9, Dako, Hamburg, Germany) before application of monoclonal antibodies specific for MSH2 (clone FE11, dilution 1:100, Calbiochem, Darmstadt, Germany), MLH1 (clone G168-15, dilution 1:100, BD Biosciences, San Diego, CA, USA), MSH6 (1:200; clone 44; BD Biosciences, San Diego, CA, USA), and PMS2 (ready to use; clone EPR3947, Cell Marque, Sigma Aldrich, St. Louis, Missouri, USA). An immunoperoxidase method was used to visualise bound antibodies with 3-amino-9-ethylcarbazole (Dako) as chromogen.

### Molecular microsatellite instability analysis

DNA was extracted using the DNeasy Blood and Tissue kit (Qiagen, Hilden, Germany) and eluted in pure water. Alternatively, cores from TMA sections were directly transferred to the polymerase chain reaction. Polymerase chain reaction with fluorescently labelled oligonucleotides was performed to amplify the mononucleotide markers BAT25, BAT26, and CAT25 from tumour tissue DNA, as described previously.^7^ Amplified fragments were visualised on an ABI3130xl genetic analyser (Applied Biosystems, Darmstadt, Germany) to detect potential length alterations of the microsatellites. Fragment sizes differing from the normal range of allelic size variation known for the amplicons encompassing BAT25 (108–110 bp), BAT26 (116–118 bp), and CAT25 (146–148 bp) were regarded as microsatellite instability.^7^ Tumours showing microsatellite instability in two or more markers were classified as microsatellite-unstable.

### Statistical analyses including correlation analyses with other immunohistochemical variables

Statistical analyses were performed with the statistical computing environment R version 3.0.1. Correlation analyses of microsatellite instability status with clinicopathological and other immunohistochemical variables were assessed with Fisher’s exact test. Due to the low number of detected microsatellite-unstable cases, a statistical correlation analysis was not feasible for all given parameters. Univariate survival analysis was performed for overall survival by generation of Kaplan-Meier curves using GraphPad Prism 6. Significance of differences between the groups was assessed using the Log-rank-test. *P* values < 0.05 were considered significant. Data concerning expression of PD-L1, Major histocompatibility Complex class I (MHC I), quantity and quality of tumour-infiltrating immune cells, as well as proliferation index (Ki-67) were available partly from previously published studies.^8,9^

## Results

### Frequency of high-level microsatellite instability in cholangiocarcinoma

**Fig. 1:**
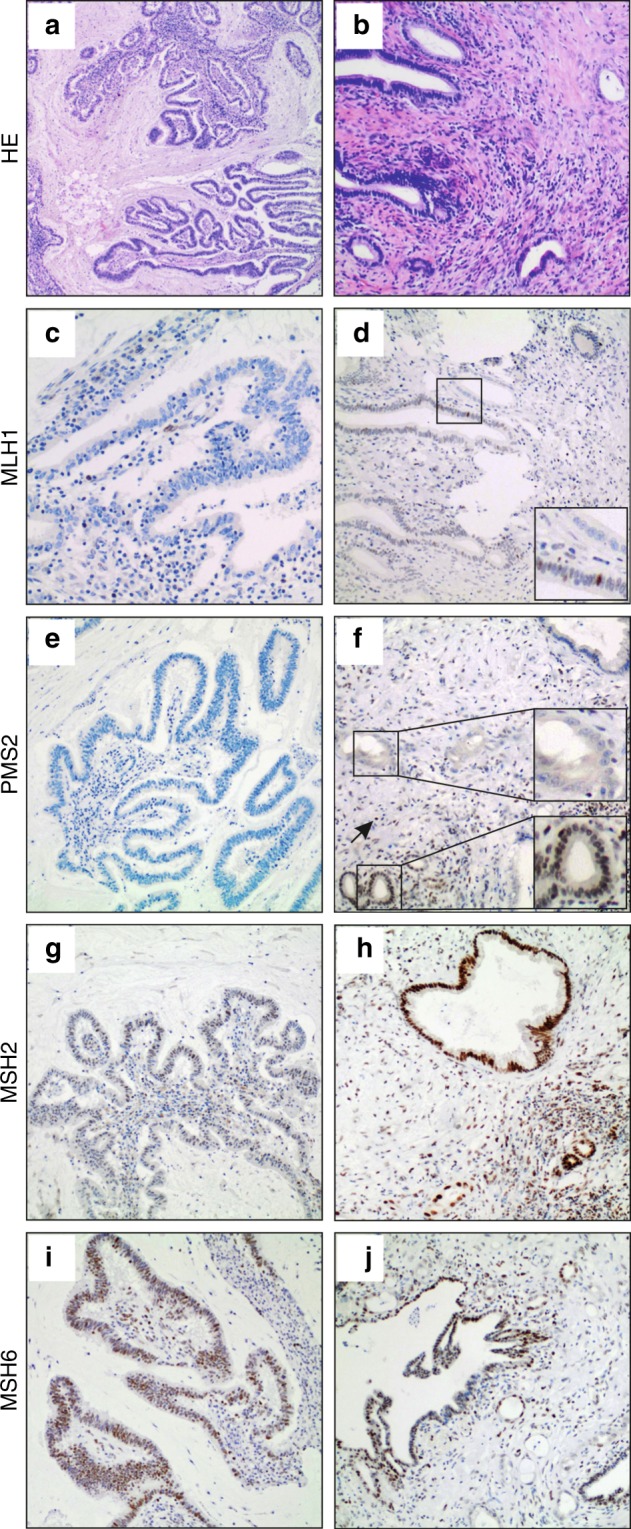
Immunohistochemistry of two representative microsatellite-unstable cases. Full slide sections of one microsatellite-unstable intrahepatic cholangiocarcinoma with papillary morphology (left sided column: **a**, **c**, **e**, **g**, **i**) showing loss of nuclear MLH1 and PMS2 immunoreactivity while MSH2 and MSH6 were retained. Full slide sections of one microsatellite-unstable perihilar cholangiocarcinoma (right sided column: **b**, **d**, **f**, **h**, **j**) showing loss of nuclear PMS2 immunoreactivity while MLH1, MSH2, and MSH6 were retained. Original magnification: 40× (H&E; **a**, **b**), 100x (**c**–**j**), 200× (insets of **d** and **f**)

A comprehensive cholangiocarcinoma cohort including all anatomical subtypes has been evaluated for microsatellite instability status. All cases were tested for microsatellite instability status using a highly sensitive mononucleotide marker panel (BAT25, BAT26, CAT25), which specifically detects DNA mismatch repair deficiency.^7,10^ Four (1.3%) out of 308 analysed cholangiocarcinomas showed high-level microsatellite instability. Of all molecular positive cases (*n* = 4), immunohistochemistry for DNA mismatch repair proteins MLH1, PMS2, MSH2, and MSH6 was performed, revealing loss of nuclear immunoreactivity of microsatellite instability markers. In detail, one microsatellite-unstable intrahepatic cholangiocarcinoma displayed loss of MLH1 and PMS2 while MSH2 and MSH6 immunoreactivity were retained (Fig. 1). The other microsatellite-unstable intrahepatic cholangiocarcinoma showed loss of MSH2 and MSH6 while MLH1 and PMS2 immunoreactivity were retained. One of the two detected microsatellite-unstable perihilar cholangiocarcinoma showed a loss of PMS2 nuclear immunoreactivity while MLH1, MSH2 and MSH6 were retained (Fig. 1). The other microsatellite-unstable perihilar cholangiocarcinoma showed loss of MSH2 and MSH6 while MLH1 and PMS2 immunoreactivity were retained. However, the immunohistochemical changes, i.e., loss of DNA mismatch repair proteins, were partly subtle, due to the histomorphological heterogeneity of cholangiocarcinoma and the mixture of non-neoplastic and neoplastic glands with partly only few evaluable neoplastic glands (e.g., Fig. 1f).

### Correlation of high-level microsatellite instability with clinicopathological data and patient survival

**Fig. 2:**
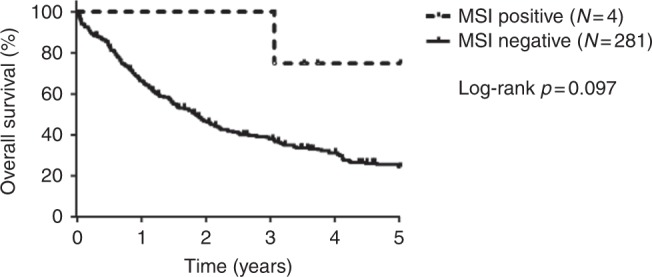
Overall survival probability in cholangiocarcinoma patients in correlation with high-level microsatellite instability status. Kaplan–Meier curves show a longer overall survival of microsatellite-unstable cholangiocarcinoma patients in correlation with microsatellite-stable cholangiocarcinoma patients (*p* = 0.097). *P*-value was calculated by log-rank test. Survival data were available for 285 of 308 (92.5%) cholangiocarcinoma patients

Analysis of cholangiocarcinoma subtypes showed that two microsatellite-unstable cases were intrahepatic (2/159, 1.3%) and two cases were perihilar extrahepatic cholangiocarcinomas (2/106, 1.9%), whereas all distal extrahepatic cholangiocarcinomas (*n* = 43) were microsatellite-stable (Table 1). Correlation with clinicopathological data showed that three of four microsatellite-unstable cases were detected in younger cholangiocarcinoma patients (younger than median patient age group: 31–63 years; Table 1). In detail, two patients were 53, one 55, and one 69 years of age. Three of four detected microsatellite-unstable patients (two intrahepatic, one perihilar cholangiocarcinomas) showed an atypical histomorphology, i.e., showing not the typical acinar/tubular/glandular/not otherwise specified pancreatobiliary phenotype, but displaying infrequent histologic patterns in cholangiocarcinoma. In detail, both microsatellite-unstable intrahepatic cholangiocarcinomas showed a predominantly papillary, partly mucinous histomorphology. One of two detected microsatellite-unstable perihilar cholangiocarcinomas had a predominantly solid and cribriform histology. The other detected microsatellite-unstable perihilar cholangiocarcinoma had the typical acinar/tubular/glandular/not otherwise specified pancreatobiliary phenotype. Furthermore, in one of the two detected microsatellite-unstable intrahepatic cholangiocarcinomas, an infrequent precursor lesion (intraductal papillary neoplasia of bile duct) was detected. This patient had Caroli syndrome, a well-established risk factor for cholangiocarcinoma. All 4 microsatellite-unstable cholangiocarcinomas showed positive lymph nodes (pN1) and a high UICC stage (UICC stage III; Table 1). Nevertheless, all cholangiocarcinoma patients with microsatellite-unstable tumours showed a longer overall survival probability when compared to patients with microsatellite-stable tumours. However, this did not reach statistical significance, probably due to the low number of detected microsatellite-unstable cholangiocarcinomas (Fig. 2; *p* = 0.097). In addition, no significant correlation with other clinicopathological data, including gender predominance or specific etiology (such as primary sclerosing cholangitis or virus hepatitis) was detected.

### Immune phenotype of microsatellite-unstable cholangiocarcinomas

We performed a correlation analysis of high-level microsatellite instability status with the quantity and quality of tumour-infiltrating immune cells, Major histocompatibility Complex class I (MHC I), and Programmed Death-Ligand 1 (PD-L1) expression in the same cholangiocarcinoma cohort.^8,9^ This revealed a significant higher number of tumour-infiltrating immune cells (particularly intratumoural CD8 + T cells, FOXP3 + regulatory T cells (Tregs), and CD20 + B cells) compared to all other microsatellite-stable cases of the cohort (*n* = 308, Table 2). In addition, three of the four detected microsatellite-unstable cases showed high or moderate Major histocompatibility Complex class I antigen expression level on the tumour cells. PD-L1 expression of the four microsatellite-unstable cases was heterogeneous: Two microsatellite-unstable cases (both intrahepatic cholangiocarcinomas) showed no PD-L1 expression, the other two microsatellite-unstable cases (both perihilar cholangiocarcinomas) showed minimal to moderate membranous and cytoplasmic PD-L1 expression (5% and 30% of all tumour cells, respectively; see supplementary figure). Additionally, we performed an integrative analysis of the immunohistochemical patterns of PD-L1 in microsatellite-unstable and microsatellite-stable CCA, showing that PD-L1 expression in tumuor cells is more common in microsatellite-unstable CCAs. However, probably due to the low number of microsatellite-unstable CCA, this finding reached only borderline statistical significance (see Supplementary table 1 and Supplementary figure 2). The proliferation index (Ki-67) was also heterogeneous (10–55%), with microsatellite-unstable cases ranking below and above the median proliferation index of microsatellite-stable cases. However, due to the low number of microsatellite-unstable cases a statistical correlation analysis was not feasible for all parameters.

**Table 2:** Comparison of microsatellite-unstabile vs microsatellite-stabile cholangiocarcinomas in regard to specific tumour-infiltrating immune cell types

All CCA patients	Total Number (%)	MSI−Number (%)	MSI+ Number(%)	*p*-value^a^
TIL cut-off value^b^	308 (100.0)	304 (98.7)	4 (1.3)	
CD4				
Intraepithelial = 0	179 (58.1)	177 (57.5)	2 (0.6)	
Intraepithelial > 0	105 (34.1)	103 (33.4)	2 (0.6)	
NA	24 (7.8)	24 (7.8)	0 (0.0)	0.62
CD8				
Intraepithelial = 0	151 (49.0)	151 (49.0)	0 (0.0)	
Intraepithelial > 0	141 (45.8)	137 (44.5)	4 (1.3)	
NA	16 (5.2)	16 (5.2)	0 (0.0)	**0.05**
FOXP3				
Total ≤ 5	219 (71.1)	218 (70.8)	1 (0.3)	
Total > 5	64 (20.8)	61 (19.8)	3 (1.0)	
NA	25 (8.1)	25 (8.1)	0 (0.0)	**0.04**
CD20				
Total = 0	164 (53.2)	164 (53.2)	0 (0.0)	
Total > 0	133 (43.2)	129 (41.9)	4 (1.3)	
NA	11 (3.6)	11 (3.6)	0 (0.0)	**0.04**

Significant values are marked in bold. ^a^Fisher’s exact test.

^b^Cut-off values were taken from ref. ^8^

## Discussion

In summary, DNA mismatch repair deficiency-induced high-level microsatellite instability status is rare in this comprehensive, well-characterised, non-liver-fluke associated German cholangiocarcinoma cohort (*n* = 308). This finding contributes to the data of other groups that showed significant differences in the pathogenesis between liver-fluke and non-liver-fluke associated cholangiocarcinoma^11–13^ that has yet not been evaluated thoroughly with regard to the role of DNA mismatch repair deficiency. In the present study, we used sensitive mononucleotide repeats, which are more specific for detecting DNA mismatch repair deficiency than di- or tetranucleotide markers,^10,14^ thereby reaching highest sensitivity of high-level microsatellite instability testing. In fact, literature studies using mononucleotide repeats for the detection of high-level microsatellite instability in cholangiocarcinoma generally reported a much lower prevalence of high-level microsatellite instability.^15–17^ All microsatellite-unstable cholangiocarcinomas could be confirmed by immunohistochemistry. As the immunohistochemical analysis of DNA mismatch repair proteins did not show a consistent pattern of expression losses of one specific protein or of protein combinations, we presume that heterogeneous molecular events are underlying mismatch repair deficiency and high-level microsatellite instability in cholangiocarcinomas.

Although the classical high-level microsatellite instability phenotype, known from microsatellite-unstable colorectal cancers was not fully recapitulated in the detected microsatellite-unstable cholangiocarcinomas, three out of four detected microsatellite-unstable cholangiocarcinomas (two intrahepatic cholangiocarcinomas and one perihilar cholangiocarcinoma) did not show the typical pancreatobiliary acinar/tubular/glandular histomorphology but displayed a papillary, mucinous or solid histologic phenotype. Interestingly, in one microsatellite-unstable intrahepatic cholangiocarcinoma, a rather infrequent precursor lesion (intraductal papillary neoplasia of the bile duct) was detected, which was present in only nine cases of the entire cohort. In addition, in this patient with intraductal papillary neoplasia of the bile duct, Caroli syndrome was also known, which is an established risk factor for cholangiocarcinoma and was present only in two patients of the entire cohort. However, the other three microsatellite-unstable cholangiocarcinomas showed no infrequent precursor lesion and were not associated with any risk factor. As the number of microsatellite-unstable cases is low in the presented cohort, these extraordinary findings in this microsatellite-unstable intrahepatic cholangiocarcinoma might be a pure coincidence. However, future studies investigating the microsatellite instability status in larger cohorts of intraductal papillary neoplasias of the bile duct and/or in Caroli syndrome-associated cholangiocarcinomas, respectively, may address the possible association of microsatellite-unstable tumours with these circumstances. In addition, no significant correlation with other clinicopathological data, including gender predominance or specific etiology (such as primary sclerosing cholangitis or virus hepatitis infection) was detected. However, the statistical correlation analysis concerning the clinicopathological data is of limited validity due to the bias of inclusion of only operated cholangiocarcinoma patients and the low number of detected microsatellite-unstable cases.

Although the overall frequency of high-level microsatellite instability in cholangiocarcinoma specimens is low with less than 2% in the present cohort, microsatellite instability testing of cholangiocarcinoma should be considered if the patients have a chance to benefit from immune checkpoint blockade.^18^ Recent studies demonstrated that patients with microsatellite-unstable cholangiocarcinomas in fact may show significant clinical responses towards treatment with anti-PD-1 or anti-PD-L1 antibodies.^1,19^ Interestingly, even cholangiocarcinoma patients with far progressed disease status that are therapy-refractory against chemotherapeutical agents commonly used in the treatment of cholangiocarcinoma can show significant treatment responses upon immune checkpoint blockade using pembrolizumab.^20^ Considering the known, pronounced and sustainable benefit reported for advanced microsatellite-unstable cancer patients after treatment with immune checkpoint inhibitors, it should be considered to test also cholangiocarcinoma patients for microsatellite instability status, despite the rarity of high-level microsatellite instability in non-liver-fluke associated cholangiocarcinogenesis. In this context, the present study underlines the significance of determining microsatellite instability in metastatic disease, demonstrating that the MSI-H phenotype as a predictor of successful immune checkpoint blockade also occurs in tumour types not typical of MMR deficiency-driven pathogenesis such as colorectal or endometrial cancer.

Importantly, microsatellite-unstable cholangiocarcinomas shared typical characteristics of microsatellite-unstable tumours of different origin, most importantly microsatellite-unstable colorectal cancers, as microsatellite-unstable cholangiocarcinomas showed a typical immune phenotype. We observed an increased number of tumour-infiltrating immune cells, features also typical of high-level microsatellite instability in colorectal cancer.^21–24^ We detected high numbers of intraepithelial CD8 + T cells, FOXP3 + regulatory T cells (Tregs), and CD20 + B cells and high/moderate Major histocompatibility Complex class I expression levels in microsatellite-unstable cholangiocarcinomas; all these parameters have been shown to be correlated with better patient survival in cholangiocarcinoma.^8,9^ The detection of enhanced expression of Major histocompatibility Complex class I antigen is compatible with an enhanced local anti-tumoural immune response in microsatellite-unstable compared to microsatellite-stable cholangiocarcinomas, although further functional studies are required to determine immune cell specificity and function in microsatellite-unstable cholangiocarcinomas.

Concerning microsatellite instability testing in cholangiocarcinoma patients, this study has several clinical implications: (i) it demonstrates that the frequency of high-level microsatellite instability is low in non-liver-fluke cholangiocarcinomas, and (ii) due to the histomorphologic heterogeneity, the use of immunohistochemistry primarily in addition to mononucleotide repeats for polymerase chain reaction-based microsatellite instability analysis seems to have highest sensitivity and specificity for the detection of DNA mismatch repair deficiency. This is in line with the situation in colorectal cancer, in which mononucleotide repeat-based microsatellite instability testing by polymerase chain reaction also has a higher sensitivity than immunohistochemical methods,^25^ however, with much smaller differences between the methods comparing to the situation in cholangiocarcinoma. (iii) microsatellite instability typing may be performed in all cholangiocarcinomas; however, high stage cholangiocarcinomas with positive nodal status, an atypical histomorphology and comparably younger patient age (< 70 years) should be primarily considered, and consequently patients with DNA mismatch repair-deficient cholangiocarcinoma may be suitable for immune checkpoint inhibition approaches.

## Electronic supplementary material

Supplemental Figure 1

Supplemental Figure 2

Supplemental Table 1

Supplementary Legends

## Availability of data and materials

All data supporting the results reported in the article can be found in the Departments of General Pathology and Applied Tumor Biology of the Institute of Pathology, University Hospital Heidelberg.

## References

[1] Le DT (2017). Mismatch-repair deficiency predicts response of solid tumors to PD-1 blockade. Science.

[2] Banales JM (2016). Expert consensus document: cholangiocarcinoma: current knowledge and future perspectives consensus statement from the European Network for the Study of Cholangiocarcinoma (ENS-CCA). Nat. Rev. Gastroenterol. Hepatol..

[3] Chan E, Berlin J (2015). Biliary tract cancers: understudied and poorly understood. J. Clin. Oncol..

[4] Nakamura H (2015). Genomic spectra of biliary tract cancer. Nat. Genet..

[5] Hause RJ, Pritchard CC, Shendure J, Salipante SJ (2016). Classification and characterization of microsatellite instability across 18 cancer types. Nat. Med..

[6] Limpaiboon T (2002). Microsatellite alterations in liver fluke related cholangiocarcinoma are associated with poor prognosis. Cancer Lett..

[7] Findeisen P (2005). T25 repeat in the 3 untranslated region of the CASP2 gene: a sensitive and specific marker for microsatellite instability in colorectal cancer. Cancer Res..

[8] Goeppert B (2013). Prognostic impact of tumour-infiltrating immune cells on biliary tract cancer. Br. J. Cancer.

[9] Goeppert B (2015). Major histocompatibility complex class I expression impacts on patient survival and type and density of immune cells in biliary tract cancer. Br. J. Cancer.

[10] Reuschenbach M (2012). Absence of mismatch repair deficiency-related microsatellite instability in non-melanoma skin cancer. J. Invest. Dermatol..

[11] Chan-On W (2013). Exome sequencing identifies distinct mutational patterns in liver fluke-related and non-infection-related bile duct cancers. Nat. Genet..

[12] Jusakul A (2017). Whole-genome and epigenomic landscapes of etiologically distinct subtypes of cholangiocarcinoma. Cancer Discov..

[13] Chaisaingmongkol J (2017). Common molecular subtypes among Asian hepatocellular carcinoma and cholangiocarcinoma. Cancer Cell..

[14] Bacher JW, Abdel Megid WM, Kent-First MG, Halberg RB (2005). Use of mononucleotide repeat markers for detection of microsatellite instability in mouse tumors. Mol. Carcinog..

[15] Liengswangwong U (2003). Infrequent microsatellite instability in liver fluke infection-associated intrahepatic cholangiocarcinomas from Thailand. Int. J. Cancer.

[16] Koo SH (2003). Microsatellite alterations in hepatocellular carcinoma and intrahepatic cholangiocarcinoma. Cancer Genet. Cytogenet..

[17] Silva VW (2016). Biliary carcinomas: pathology and the role of DNA mismatch repair deficiency. Chin. Clin. Oncol..

[18] Pardoll DM (2012). The blockade of immune checkpoints in cancer immunotherapy. Nat. Rev. Cancer.

[19] Le DT (2015). PD-1 blockade in tumors with mismatch-repair deficiency. N. Engl. J. Med..

[20] Czink E (2017). Successful immune checkpoint blockade in a patient with advanced stage microsatellite unstable biliary tract cancer. Cold Spring Harb. Mol. Case Stud.

[21] Shia J (2003). Value of histopathology in predicting microsatellite instability in hereditary nonpolyposis colorectal cancer and sporadic colorectal cancer. Am. J. Surg. Pathol..

[22] Dolcetti R (1999). High prevalence of activated intraepithelial cytotoxic T lymphocytes and increased neoplastic cell apoptosis in colorectal carcinomas with microsatellite instability. Am. J. Pathol..

[23] Bauer K (2011). Dendritic cell and macrophage infiltration in microsatellite-unstable and microsatellite-stable colorectal cancer. Fam. Cancer.

[24] Mlecnik B (2016). Integrative analyses of colorectal cancer show immunoscore is a stronger predictor of patient survival than microsatellite instability. Immunity.

[25] Lindor NM (2002). Immunohistochemistry versus microsatellite instability testing in phenotyping colorectal tumors. J. Clin. Oncol..

