# Supplementary figures and images for: Mismatch repair deficiency is a rare but putative therapeutically relevant finding in non-liver fluke associated cholangiocarcinoma
Source: Br J Cancer. 2018 Oct 31;120(1):109–14. doi: 10.1038/s41416-018-0199-2 (PMC6325153; doi:10.1038/s41416-018-0199-2)

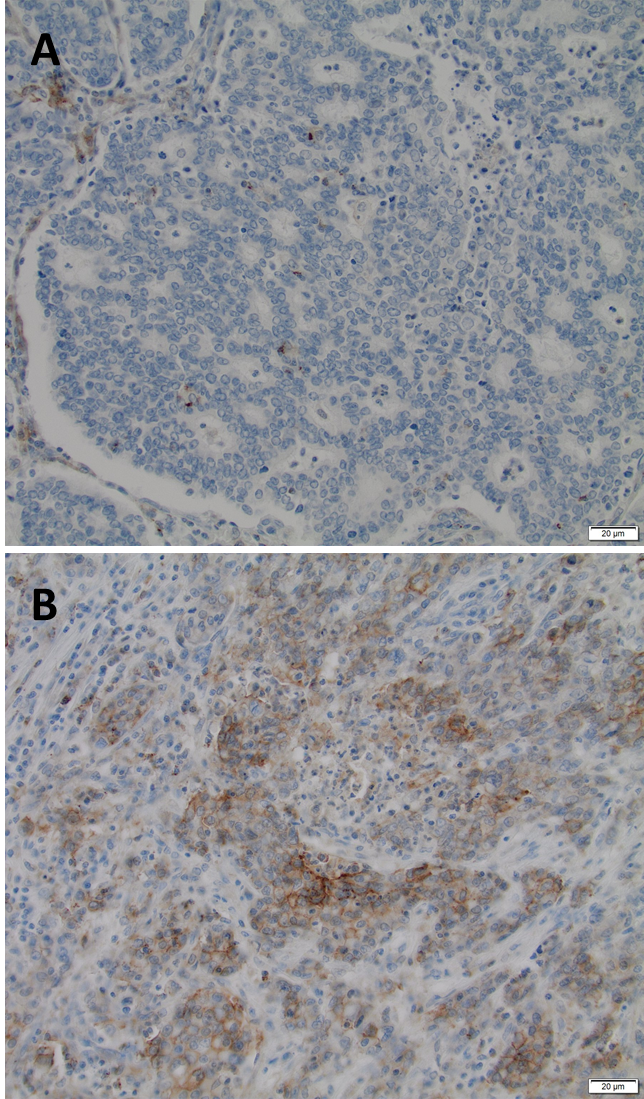

Supplement: Supplementary file 1 — Supplemental Figure 1 [file 41416_2018_199_MOESM1_ESM.tif]

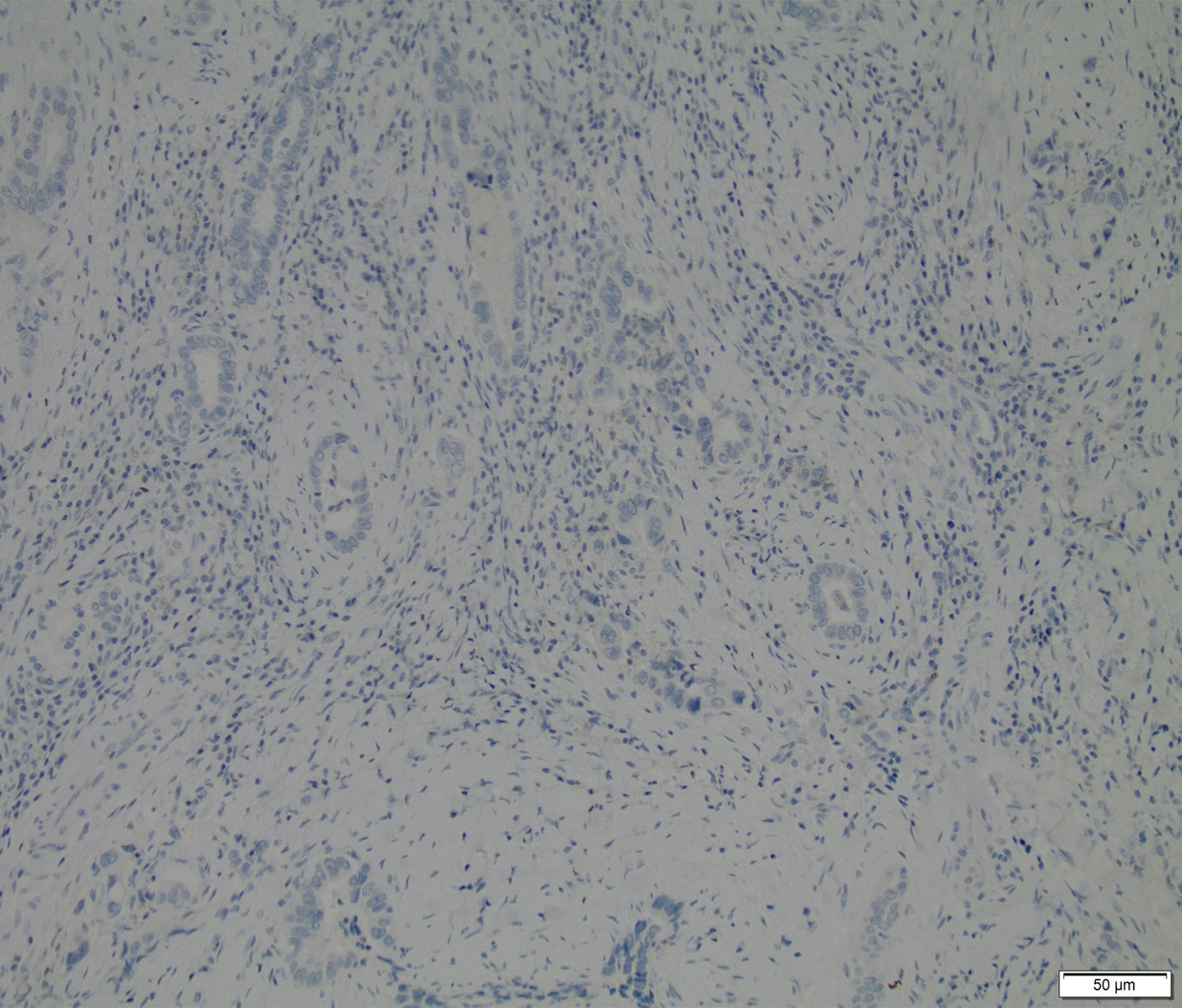

Supplement: Supplementary file 2 — Supplemental Figure 2 [file 41416_2018_199_MOESM2_ESM.tif]
