# Supplementary material for: Mismatch repair deficiency is a rare but putative therapeutically relevant finding in non-liver fluke associated cholangiocarcinoma
Source: Br J Cancer. 2018 Oct 31;120(1):109–14. doi: 10.1038/s41416-018-0199-2 (PMC6325153; doi:10.1038/s41416-018-0199-2)
Supplement: Supplementary file 4 — Supplementary Legends [file 41416_2018_199_MOESM4_ESM.docx]

**Supplemental Figure 1:** PD-L1 immunohistochemistry of two representative microsatellite-unstable cases.

PD-L1 immunohistochemistry performed on full slide sections of one microsatellite-unstable intrahepatic cholangiocarcinoma with tubulopapillary morphology with PD-L1-negative tumour cells and some PD-L1 positive immune cells (A; original magnification: 200x) and one extrahepatic cholangiocarcinoma showing PD-L1 positive tumour cells (B; original magnification: 200x).

**Supplemental Figure 2:** PD-L1 immunohistochemistry of a representative microsatellite-stable CCA showing no PD-L1 expression on tumor cells.

PD-L1 immunohistochemistry performed on full slide sections of one microsatellite-stable extrahepatic cholangiocarcinoma with glandular/tubular/acinar morphology with PD-L1-negative tumour cells and few PD-L1 positive immune cells in the tumor stroma (original magnification: 100x).

**Supplemental Table 1:** Correlation of PD-L1 status with MSI status.

PD-L1 immunohistochemistry was available for a subset (n=144) of the CCA cohort. Statistical significance was determined by Fisher's exact test: p=0.084.
